# Outcome measures for oral health based on clinical assessments and claims data: feasibility evaluation in practice

**DOI:** 10.1186/s12903-017-0410-5

**Published:** 2017-10-05

**Authors:** Riët Hummel, Josef Bruers, Onno van der Galiën, Wil van der Sanden, Geert van der Heijden

**Affiliations:** 1 Department of Social Dentistry, Academic Centre for Dentistry Amsterdam (ACTA), University of Amsterdam and Vrije Universiteit Amsterdam, Gustav Mahlerlaan 3004, 1081 LA Amsterdam, the Netherlands; 2 Zilveren Kruis Achmea, Leusden, the Netherlands; 3 KNMT, Royal Dutch Dental Association, Nieuwegein, the Netherlands; 4 Department of Quality and Safety of Oral Health Care, College of Oral Science, Radboud University Medical Center, Nijmegen, The Netherlands

**Keywords:** Outcome assessment health care, Quality measures, Quality improvement, Oral health, Dental caries, Physician’s practice patterns

## Abstract

**Background:**

It is well known that treatment variation exists in oral healthcare, but the consequences for oral health are unknown as the development of outcome measures is still in its infancy. The aim of this study was to identify and develop outcome measures for oral health and explore their performance using health insurance claims records and clinical data from general dental practices.

**Methods:**

The Dutch healthcare insurance company Achmea collaborated with researchers, oral health experts, and general dental practitioners (GDPs) in a proof of practice study to test the feasibility of measures in general dental practices. A literature search identified previously described outcome measures for oral healthcare. Using a structured approach, identified measures were (i) prioritized, adjusted and added to after discussion and then (ii) tested for feasibility of data collection, their face validity and discriminative validity. Data sources were claims records from Achmea, clinical records from dental practices, and prospective, pre-determined clinical assessment data obtained during routine consultations.

**Results:**

In total eight measures (four on dental caries, one on tooth wear, two on periodontal health, one on retreatment) were identified, prioritized and tested. The retreatment measure and three measures for dental caries were found promising as data collection was feasible, they had face validity and discriminative validity. Deployment of these measures demonstrated variation in clinical practices of GDPs. Feedback of this data to GDPs led to vivid discussions on best practices and quality of care. The measure ‘tooth wear’ was not considered sufficiently responsive; ‘changes in periodontal health score’ was considered a controversial measure. The available data for the measures ‘percentage of 18-year-olds with no tooth decay’ and ‘improvement in gingival bleeding index at reassessment’ was too limited to provide accurate estimates per dental practice.

**Conclusions:**

The evaluated measures ‘time to first restoration’, ‘distribution of risk categories for dental caries’, ‘filled-and-missing score’ and ‘retreatment after restoration’, were considered valid and relevant measures and a proxy for oral health status. As such, they improve the transparency of oral health services delivery that can be related to oral health outcomes, and with time may serve to improve these oral health outcomes.

**Electronic supplementary material:**

The online version of this article (doi:10.1186/s12903-017-0410-5) contains supplementary material, which is available to authorized users.

## Background

The aim of oral healthcare is to maintain or improve oral health for individuals and populations [1]. The degree to which the likelihood of this goal is increased by the delivered oral healthcare services is regarded as quality of care [2]. Quality of care can therefore be explored using data which describes links between the healthcare services provided and improved oral health outcomes. These may comprise both clinical and non-clinical, patient-derived outcomes. Transparency on health services delivered may be a helpful tool in reducing unwarranted treatment variation [3] and is therefore of increasing interest in today’s society [1].

Information on delivered health services may provide patients with expectations about their care, and can be used by insurance companies and other policy makers in assessing the balance between the outcomes and costs of care. In addition, it may provide general dental practitioners (GDPs) with insight into their clinical activities compared to their peers, which they could potentially use for reflection and, if required, for care quality improvement.

Use of clinical activity data with the aim of improving health outcomes is not the norm in oral healthcare [4]. Development of measures is limited by the few evidence based guidelines available, their variable implementation, and the lack of consensus on what is the best oral care for most conditions and situations [1, 4, 5]. It is therefore not surprising that variation in clinical practice has been described [1, 4, 6, 7].

A number of initiatives on quality measures and indicators for oral health and healthcare service delivery are reported, mainly in Anglo-Saxon and Scandinavian countries [8–16]. They have compiled lists of performance measures. These lists show considerable overlap, while the topics included are often poorly defined and their development has often not been completed [14, 17]. Only a few articles described the development of oral healthcare delivery topics into measures and reported on their performance [6, 17, 18]. Apart from Herndon et al. [6], the authors did not fully specify the measures, nor did they report data per dental practice, which hampers reproducibility and limits their usability. Worldwide, the development of quality measures for oral healthcare is still in its infancy [1, 17].

Achmea, one of the major health insurance companies in the Netherlands with more than four million insured persons, therefore has explored the measurement of oral health outcomes with a group of researchers, advising experts and five general dental practices. The aim was to select and develop clinical outcome measures for oral health and provided oral health services that are considered relevant, valid and important in oral health, in the context of using clinical data from general dental practices and claims records of an insurance company. We used a structured approach to select and derive these measures and to evaluate them in practice by assessing the feasibility of data collection, face validity and discriminative validity.

## Methods

### Selection and development of measures

MEDLINE-PubMed was searched (on Dec 3, 2012) to identify outcome measures for oral healthcare published in the previous 5 years. Titles and abstracts of papers identified in the search were scanned to select papers that potentially included outcome measures for oral health. In addition, we searched the databases of relevant Dutch oral healthcare organizations and international organizations involved in the production of quality measures for healthcare. The MEDLINE-PubMed search strategy and organizations are listed in Table 1.

**Table 1:** Identification of described outcome measures in oral healthcare. Contains the MEDLINE-PubMed search strategy and a list of databases and organizations that were searched

MEDLINE-PubMed search
MeSH terms: ‘dental care’ or ‘quality indicators, health care’ Title or abstract words: ‘dental care’, ‘dental service’, ‘quality indicator’, ‘outcome indicator’, ‘performance measure’ or ‘outcome measure’
Dutch organizations, not specifically aimed at guidelines and measures
www.iqhealthcare.nl
https://www.regioplan.nl/publicaties/rapporten/operationaliseren_kwaliteitsindicatoren_mondzorg_1
www.centrumklantervaringzorg.nl
http://www.patientuitkomsten.nl/vragenlijst/voorbeeld-vragenlijsten*
www.rivm.nl
www.nivel.nl
www.onderzoekinformatie.nl
www.oncoline.nl
http://www.med-info.nl
https://www.knmt.nl/richtlijnen-mondzorg
Organizations specialized in quality measures
www.oecd.org
www.nice.org.uk
http://ichom.org
www.aezq.de
www.qualitymeasures.ahrq.gov/browse/by-topic.aspx
www.aqua-institut.de
www.bqs-online.de
www.cms.hhs.gov
www.ama-assn.org
www.jcaho.org
www.ncqa.org
https://www.facs.org/quality-programs/acs-nsqip
www.pressganey.com
http://www.qualityforum.org
www.achs.org.au
www.internationalqip.com/indicators.aspx*
www.productivity.nhs.uk
http://www.scot.nhs.uk/
http://www.nhs.uk
https://www.gov.uk/government/organisations/healthcare-commission
www.drfosterintelligence.co.uk/
http://hpm.org/en/Surveys/University_of_Southern_Denmark_-_Denmark/12/The_National_Indicator_Project.html
www.oha.com
www.ccort.ca
www.cancercare.on.ca
www.who.int/en/
http://www.rand.org/
www.tumori.net/eurochip
http://ec.europa.eu/
www.guidelines.gov
www.nursingworld.org
www.cigna.com
www.aetna.com
http://drc.hhs.gov/indicators.htm

*The search was performed in 2012. This URL was active in 2012, but no longer accessible in 2017

Four oral healthcare experts were invited to participate in the project advisory board. These experts were identified by their work on quality of care topics in Dutch dental schools and professional associations for dentists. They assisted in the selection and definition of measures by prioritizing the identified measures and judging whether the list was comprehensive, and suggesting additional measures. Subsequently they selected measures on the following criteria: relevance for quality improvement for professionals in daily oral healthcare practice; reliability; validity; and limited administrative burden. The experts discussed their findings with Achmea project staff.

 This process resulted in a set of four measures related to dental caries and its treatment, one measure on tooth wear, two on periodontal health and one measure on retreatment after restoration. The selected measures were then precisely defined, and methods of measurement and data collection were determined. The specifications are listed under the heading ‘specifications of measure’ in Tables 2, 3, 4, 5, 6, 7 and Table 8.

**Table 2:** ‘Time to first restoration’: patient characteristics, results, specifications

Patient characteristics and results per dental practice (DP)					
*Patients*	*DP #1*	*DP #2*	*DP #3*	*DP #4*	*All DPs*
Per socioeconomic status					
- High	27%	64%	85%	64%	58%
- Middle	12%	8%	9%	6%	9%
- Low	60%	27%	5%	29%	33%
- Unknown		1%	1%	1%	1%
n	179	109	131	181	600
*Results, patients*					
Restoration-free on the age of:					
- 2 years	99%	100%	100%	100%	100%
- 3 years	97%	99%	100%	99%	99%
- 4 years	87%	94%	95%	92%	91%
- 5 years	73%	84%	91%	79%	81%
- 6 years	53%	72%	81%	66%	66%
- 7 years	42%	65%	76%	60%	59%
There was a difference in restoration-free seven-year-olds between the dental practices (*p* < 0.01, Chi-square test). Overall, there was a difference in restoration-free seven-year-olds between the socioeconomic groups in all dental practices (*p* < 0.01, Chi-square test); in the highest socioeconomic group there were more children with a restoration-free dentition and less in the lowest socioeconomic group.					
Specifications of measure					
Data source	Clinical records dental practices				
Assessment based on	Restorations				
Inclusion criteria	• Children born between July 1st 2006 and July 1st 2007 (7-year-olds) • Registered in the dental practice between 2010 and 2014 • At least one oral health examination a year • No caries during the first visit and the first half year after the first visit • Primary teeth

**Table 3:** ‘Distribution of risk categories for dental caries based on clinical assessment’: patient characteristics, results, specifications

Patient characteristics and results per dental practice (DP), children and adolescents (<18 years)					
*Patients < 18 years old*	*DP #1*	*DP #2*	*DP #3*	*DP #4*	*All DPs*
Per age group					
- 0–6 years	18%	21%	24%		21%
- 7–12 years	43%	39%	52%		45%
- 13–17 years	39%	40%	24%	100%	33%
Per socioeconomic status					
- High	23%	77%	88%	33%	70%
- Middle	9%	5%	1%		4%
- Low	68%	17%	6%	67%	24%
- Unknown		1%	5%		3%
n	77	115	153	3	348
*Results, patients < 18 years old*					
Per risk category, baseline					
- Low	38%	17%	69%	100%	45%
- Decreased	12%	22%	19%		18%
- Increased	22%	28%	10%		18%
- High	29%	34%	3%		19%
Patient characteristics and results per dental practice, adults (≥18 years)					
*Patients ≥ 18 years old*	*DP #1*	*DP #2*	*DP #3*	*DP #4*	*All DPs*
Per age group					
- 18–39	51%	41%	52%	44%	48%
- 40+	49%	59%	48%	56%	52%
Per socioeconomic status					
- High	33%	70%	83%	68%	62%
- Middle	17%	9%	4%	5%	9%
- Low	51%	19%	5%	24%	25%
- Unknown		3%	9%	3%	4%
n	150	153	149	66	518
*Results, patients ≥ 18 years old*					
Per risk category, baseline					
- Low	8%	5%	6%	26%	9%
- Decreased	26%	44%	74%	20%	44%
- Increased	33%	41%	16%	26%	30%
- High	33%	10%	4%	29%	17%
For children and adolescents the distribution of risk categories for dental caries varied between the dental practices (*p* < 0.01, Kruskal-Wallis test). The caries risk was highest in dental practice #2 and lowest in dental practice #3. Overall, there was a difference in the distribution of risk categories in the different age groups (*p* < 0.01, Kruskal-Wallis test). Children in age group 0–6 years were in lower risk categories than children in age group 7–12 and adolescents in age group 13–17 years. There was a difference in distribution of risk categories in the different socioeconomic groups (*p* < 0.01, Kruskal-Wallis test). Children and adolescents in the lowest socioeconomic group were in higher risk categories than children and adolescents in the middle and low group. For adults the distribution of risk categories for dental caries also varied between the dental practices (*p* < 0.01, Kruskal-Wallis test). The caries risk was highest in dental practice #1 and lowest in dental practice #3. Overall, there was a difference in the distribution of risk categories in both age groups (*p* < 0.05 Mann-Whitney U-test). Adults in age group 40+ were in higher risk categories than adults in age group 18–39 years. There was a difference in distribution of risk categories in the different socioeconomic groups (*p* < 0.01, Kruskal-Wallis test). Adults in the highest socioeconomic group were in lower risk categories than adults in the middle and low group.					
Specifications of measure					
Data source	Clinical assessment by the GDP during consultation				
Assessment of risk categories	*Low* - No restorations and no active caries lesions in the past *Decreased *- Restorations or lesions in the past, but not in the last year *Increased* - Active lesions and 1 new or progressed lesion in the last year *High* - Active lesions and 2 or more new and/or progressed lesions in the last year [26]				
Numerator/denominator	Numerator: number of patients per risk category Denominator: total number of patients with an assessment of a risk category				
Inclusion criteria	• at least one oral health examination a year • at least 2 years registered in the dental practice

**Table 4:** ‘Distribution of risk categories for dental caries based on claims records insurance company’: patient characteristics, results, specifications

Results per dental practice (DP), children and adolescents (<18 years)					
*Patients < 18 years old*	*DP #1*	*DP #2*	*DP #3*	*DP #4*	*All DPs*
n	205	105	502	412	1224
Per risk category, baseline					
- Low	39%	59%	71%	63%	62%
- Decreased	18%	21%	13%	15%	15%
- Increased	13%	13%	10%	13%	12%
- High	30%	7%	6%	9%	11%
Results per dental practice adults (≥18 years)					
*Patients ≥ 18 years old*	*DP #1*	*DP #2*	*DP #3*	*DP #4*	*All DPs*
n	544	114	725	614	1997
Per risk category, baseline					
- Low	26%	21%	30%	22%	26%
- Decreased	31%	34%	31%	21%	28%
- Increased	18%	20%	22%	26%	22%
- High	25%	25%	17%	32%	24%
For children and adolescents as well as adults the distribution of risk categories for dental caries for all dental practices is not the same when based on clinical assessments and claims records (*p* < 0.01, respectively *p* < 0.05, Mann-Whitney U-test). Based on clinical assessments the caries risk is higher than based on claims records. However when for adults both lowest and both highest risk categories were merged the distribution is the same between both data sources (*p* > 0.05, Chi-square test).					
Specifications of measure					
Data source	Claims records insurance company				
Assessment of risk categories	*Low* - No known restorations in claims records insurance company in the last 3 years *Decreased* - No restorations in the last year, but 1 or more in the 2 years before last year *Increased* - 1 restoration in the last year *High* - 2 or more restorations in the last year (in different teeth) Modified from ‘distribution of risk categories for dental caries based on clinical assessment’ [26]				
Assessment based on	• Restorations • Slicing and/or treatment of primary teeth • Stainless steel crowns primary teeth

**Table 5:** ‘Filled-and-Missing (FM) score’: patient characteristics, results, specifications

Patient characteristics and results per dental practice (DP)					
*Patients*	*DP #1*	*DP #2*	*DP #3*	*DP #4*	National*)
Per age group					
- 0–6 years	18%	15%	27%	13%	17%
- 7–12 years	47%	46%	52%	52%	51%
- 13–17 years	35%	38%	22%	35%	32%
Per socioeconomic status					
- High	23%	71%	91%	71%	65%
- Middle	11%	5%	3%	4%	8%
- Low	66%	24%	6%	25%	28%
n	376	387	602	574	234,033
*Results*					
FM-score (X; SD)	1.5 (2.32)	0.6 (1.31)	0.3 (0.77)	0.6 (1.22)	0.6 (1.29)
Restoration score (X; SD)	1.34 (2.13)	0.46 (1.06)	0.25 (0.70)	0.51 (1.12)	0.49 (1.16)
Extraction score (X;XD)	0.16 (0.63)	0.14 (0.64)	0.07 (0.32)	0.09 (0.36)	0.11 (0.50)
*) National is based on all the claims records from Achmea for dental practices with more than 50 young patients that are insured by Achmea.					
The differences in FM-score between the dental practices were statistically significant (*p* < 0.01, Kruskal-Wallis test). Overall, differences in FM-score between the different socioeconomic groups were statistically significant (*p* < 0.01, Kruskal-Wallis test). The FM-score is lower for children and adolescents in the highest socioeconomic group compared to the middle and low groups. Differences in FM-score between age groups were not considered clinically relevant.					
Specifications of measure					
Data source	Claims records insurance company				
Assessment based on	• Restorations • Slicing and/or treatment of primary teeth • Stainless steel crowns primary teeth • Extractions				
Formula FM-score	**Restoration (F) score:** [(number of children and adolescents with restorations/total number of children and adolescents with at least one dental examination) x (number of restorations/number of children and adolescents with restorations)] + **Extraction (M) score:** [(number of children and adolescents with extractions/number of children and adolescents with at least one dental examination) x (number of extractions/number of children with extractions)]				
Inclusion criteria	• children and adolescents <18 years old • at least one oral health examination a year • at least 2 years registered in the dental practice • exclusion of extractions on behalf of orthodontic treatment (multiple extractions of premolars) and wisdom teeth

**Table 6:** ‘Retreatment after restoration’: numbers, results, specifications

Numbers and results per dental practice (DP), children and adolescents (<18 years)				
*Patients < 18 years old*	*DP #1*	*DP #2*	*DP #3*	*DP #4*
Number of restorations	614	220	88	480
*Results patients < 18 years old, teeth with*				
Re-restoration within				
- 6 months	(0)	(0)	8% (0.27)	(0)
- 12 months	0.5% (0.07)	(0)	11% (0.32)	(0)
- 18 months (SD)	1% (0.11)	1% (0.12)	15% (0.36)	(0)
Endodontic treatment within 6 months after restoration	(0)	(0)	(0)	(0)
Extraction within 6 months after restoration	(0)	(0)	(0)	(0)
Patient characteristics and results per dental practice, adults (≥18 years)				
*Patients ≥ 18 years old*	*DP #1*	*DP #2*	*DP #3*	*DP #4*
Number of restorations	5434	2671	2007	7389
*Results patients ≥ 18 years old, teeth with*				
Re-restoration within				
- 6 months	(0.05)	1% (0.08)	2% (0.14)	(0.06)
- 12 months	(0.07)	1% (0.10)	4% (0.20)	1% (0.09)
- 18 months (SD)	1% (0.09)	2% (0.14)	7% (0.25)	2% (0.13)
Endodontic treatment within 6 months after restoration	(0.03)	(0.02)	(0.05)	(0.06)
Extraction within 6 months after restoration	(0.03)	(0.03)	(0.05)	(0.04)
For children and adolescents there was a difference in re-restorations within 18 months between the dental practices (*p* < 0.01, Chi-square test). Dental practice #3 had more re-restorations and dental practice #4 less. For adults there was a difference in re-restoration within 18 months between the dental practices (*p* < 0.01, Chi-square test). Dental practice #3 had more re-restorations and dental practice #1 less.				
Specifications on measure				
Data source	Clinical records dental practice			
Assessment based on	• Restorations • Extractions • Endodontic treatment			
Numerator/denominator	Numerator: a) number of teeth that had a re-restoration within respectively 6, 12 and 18 months b) number of teeth with an endodontic treatment within 6 months after restoration c) number of teeth that were extracted within 6 months after restoration Denominator: number of teeth that were restored in the year 2012			
Inclusion criteria	• Patient is registered in the dental practice during the entire 18 months • Permanent teeth

**Table 7:** ‘Tooth wear’: patient characteristics, results, specifications

Patient characteristics and results per dental practice (DP), children and adolescents (<18 years)					
*Patients < 18 years old*	*DP #1*	*DP #2*	*DP #3*	*DP #4*	*All DPs*
Per age group					
- 7–12 years	18%	12%	9%		12%
- 13–17 years	82%	88%	91%	100%	88%
Per socioeconomic status					
- High	24%	78%	94%	33%	70%
- Middle	12%	6%	2%		6%
- Low	65%	16%	4%	67%	24%
n	34	49	53	3	139
*Results, patients < 18 years old*					
Per score *vertical* tooth wear					
- 0	41%	49%	42%	100%	45%
- 1	59%	47%	55%		52%
- 2		4%	4%		3%
- 3					
- 4					
Per score *horizontal* tooth wear					
- 0	68%	82%	100%	100%	86%
- 1	32%	12%			12%
- 2		6%			2%
Patient characteristics and results per dental practice, adults (≥18 years)					
*patients ≥ 18 years old*	*DP #1*	*DP #2*	*DP #3*	*DP #4*	*All DPs*
Per age group					
- 18–39	53%	45%	54%	49%	50%
- 40–64	47%	55%	46%	51%	50%
Per socioeconomic status					
- High	35%	71%	91%	70%	62%
- Middle	15%	10%	4%	5%	13%
- Low	50%	19%	5%	25%	25%
n	131	133	139	61	464
*Results, patients ≥ 18 years old*					
Per score *vertical* tooth wear					
- 0	17%	23%	2%	16%	14%
- 1	74%	30%	62%	44%	54%
- 2	9%	47%	34%	36%	31%
- 3			2%	3%	1%
- 4					
Per score *horizontal* tooth wear					
- 0	62%	83%	40%	84%	64%
- 1	34%	12%	17%	11%	20%
- 2	4%	5%	43%	5%	16%
Differences between dental practices were visible, but they were mainly explained by differences in the way tooth wear was assessed. The GDPs experienced difficulties in the use of the assessment instrument and used it differently because the definition of the instrument was not clear enough. Therefore the results of the statistical tests are not reported.					
Specifications of the measure					
Data source	Clinical assessment by the GDP during consultation				
Clinical assessment based on	*Vertical tooth wear*: score per sextant the amount of lost tooth material on the occlusal/incisal surfaces [27] 0 – no (visible) tooth wear 1 – visible tooth wear only in enamel 2 – exposed dentin and loss of clinical crown height < 1/3 3 – loss of clinical crown height > 1/3, but <2/3 4 – loss of clinical crown height > 2/3 *Horizontal tooth wear*: score per sextant the amount of lost tooth material on the not occlusal/not incisal surfaces [27] 0 – no (visible) tooth wear 1 – visible tooth wear only in enamel 2 – exposed dentin				
Numerator/denominator	To be defined. Due to the limited time of the study only baseline measures were conducted.				
Inclusion criteria	• at least one oral health examination a year • at least 2 years registered in the dental practice • permanent teeth

**Table 8:** ‘Changes in periodontal-health score’: patient characteristics, results, specifications

Patient characteristics and results per dental practice (DP)					
*Patients*	*DP #1*	*DP #2*	*DP #3*	*DP #4*	*All DPs*
Per age group					
- 18–39 years	50%	41%	48%	30%	45%
- 40–64 years	49%	52%	50%	57%	51%
- 64+	1%	7%	2%	13%	4%
Per socioeconomic status					
- High	31%	73%	90%	70%	66%
- Middle	15%	9%	5%	4%	9%
- Low	54%	18%	6%	26%	25%
Smoking	10%	7%	16%	26%	12%
With diabetes	3%	1%	3%	4%	2%
n	102	138	105	23	368
*Results, patients*					
Per index category DPSI category A *)					
- 0		2%			1%
- 1	33%	1%	20%	4%	16%
- 2	9%	31%	64%	39%	35%
DPSI category B					
- 3-	47%	46%	11%	48%	37%
- 3+	3%	1%			1%
DPSI category C					
- 4	8%	17%	5%	9%	11%
With an					
- Improved DPSI index	28%	20%	28%	17%	24%
- Impaired DPSI index	17%	18%	18%	17%	18%
Moved from					
- DPSI category C to A/B	7%	7%	1%		5%
- DPSI category A/B to C	4%	4%	2%		3%
The distribution of DPSI-indices varied between the dental practices (*p* < 0.01, Kruskal-Wallis test). The DPSI is the lowest in dental practice #3. There was no difference in patients moving from DPSI category C to category A or B between the dental practices (*p* > 0.05 Chi-square test), nor for patients moving from DPSI category A or B to category C (*p* > 0.05, Chi-square test). The DPSI of patients in age group 40–64 years was higher than of patients in age category 18–39 years (*p* < 0.01, Mann-Whitney U-test). There was no difference in DPSI between the different socioeconomic groups (*p* > 0.05, Kruskal-Wallis test). Smokers and non-smokers have the same distribution of DPSI-indices (*p* > 0.05, Mann-Whitney U-test). This is also the case for patients with and without diabetes (*p* > 0.05, Mann-Whitney U-test).					
Specifications of measure					
Data source	Clinical records dental practice				
*) Assessment of DPSI	The DPSI-index is the highest measured score for measures in all six sextants [28] *DPSI category A* DPSI index 0 - no pockets deeper than 3 mm, no bleeding on probing, no calculus, no overhangs of restorations DPSI index 1 - same as in index 0, but bleeding on probing DPSI index 2 - same as in index 1, but with calculus and/or overhanging restorations *DPSI category B* DPSI index 3 negative - pockets of 4 or 5 mm with bleeding on probing WITHOUT gingival recession(s) above the deepened pocket(s) *DPSI category C* DPSI index 3 positive - pockets of 4 or 5 mm with bleeding on probing WITH gingival recession(s) above the deepened pocket(s) DPSI index 4 - one or more pockets of at least 6 mm in depth				
Numerator/denominator	Numerator: a) number of patients that moved from DPSI-category A or B to category C b) number of patients that moved from DPSI-category C to A or B at reassessment Denominator: total number of patients with a repeated assessment of the DPSI-score				
Inclusion criteria	• at least one oral health examination a year • at least 2 years registered in the dental practice • ≥18 years old				

### Measures

#### Dental caries

##### Time to first restoration

The time to the first restoration is a proxy for the time of good oral health until the first, irreversible, invasive dental intervention. The longer this time interval, the more likely it is that effective preventive healthcare and preventive self-care is provided.

##### Distribution of risk categories for dental caries based on clinical assessment

The clinically assessed risk categories were based on new carious lesions and progressed existing carious lesions. This included carious lesions with an indication for restorative treatment as well as active initial lesions without an indication for restorative treatment. The definition of the four risk categories (low, decreased, increased and high) are described in Table 3.

##### Distribution of risk categories for dental caries based on claims records

Assessment of the dental caries risk categories using claims records was based on claimed restorations. The definition of the categories (low, decreased, increased, high) are described in Table 4.

##### Filled-and-missing (FM) score

The FM-score is the average number of restorations plus extractions per child or adolescent per dental practice per year, a proxy for the increase in decayed, missing and filled permanent or primary teeth or both (ΔDMFT/Δdmft). Preservation of tooth material is an aspect of oral health and every restoration, re-restoration and extraction means loss of tooth material.

##### Percentage of 18-year-olds with no tooth decay

This measure reflects the outcomes of preventive efforts from both patients and the oral healthcare team until adulthood.

#### Tooth wear

Percentage of patients per category for vertical (occlusal/incisal) and horizontal (not occlusal/incisal) tooth wear. Tooth wear is irreversible, but the process can be stopped or slowed. Assessment, recording and monitoring is necessary to be able to intervene in an early stage before extensive restorative treatment is required.

#### Periodontal health

##### Changes in periodontal-health score

Periodontal health is evaluated during the oral health examination using the Dutch Periodontal Screening Index (DPSI). DPSI is the Community Periodontal Index of Treatment Needs (CPITN), modified for the Netherlands. This measure shows the percentage of patients moving from DPSI category A (minor periodontal disease) or B (moderate periodontitis) to DPSI category C (severe periodontitis) and vice versa [19]. Prevention and treatment should aim on keeping patients out of the irreversible danger zone (DPSI category C).

##### Improvement in gingival bleeding index at reassessment

Percentage DPSI category B and C patients whose gingival bleeding index is lower at reassessment after initial periodontal treatment. Improvement of the DPSI-index is not always achievable in patients with periodontitis. DPSI-B or DPSI-C at reassessment indicates that there is at least one remaining site with a pocket, while most of the other pockets and inflammations may have been eliminated. The bleeding index is an additional measure to assess improvement in periodontal health.

#### Retreatment

Retreatment is the percentage of teeth with a re-restoration within 6, 12 and 18 months after restoration. It also measures the percentage of endodontically treated and extracted teeth within 6 months after restoration. The measure provides information about the success rate and complications after restorative treatment in comparison with other dental practices. It can reflect incorrect indications, unforeseen complications, or failures of the restorations. Variation can also result from differences in clinical management of the same condition by different GDPs.

### Participants

Five GDPs who expressed an interest in the quality of care improvement project in conversations with Achmea were invited to participate in the project. They all worked in group practices with several GDPs per practice with a range of two to seven full-time or part-time working GDPs. From the five participating GDPs, one did not participate in the data collection and another did not participate in the discussion and interpretation of data. So, three GDPs participated throughout all project phases.

The socioeconomic status was generally high in three of the four dental practices that participated in the data collection. The socioeconomic status of the patients of the other dental practice was mainly low. Patient characteristics from the participating GDPs are described per measure in Tables 2, 3, 4, 5, 6, 7 and 8.

### Data sources

Data sources were claims data for provided care (fee per item), clinical records for information on oral health status or more detailed information on provided care, and assessment during consultations for not regularly recorded information on oral health. Since Achmea data only covers part of the patient population of GDPs, clinical records were also used in measures with limited numbers of patients. The clinically assessed measures only used data from patients examined by the participating GDPs; the measures based on patients or claims data could also comprise care provided by other GDPs working in the dental practices.

Data on health insurance claims for selected measures were extracted from the Achmea records for the period January 2011 through December 2013. Data from the clinical records were extracted from the patient files of GDPs for the period January 2009 through December 2014. Clinical assessment data were collected by the GDPs from May through October 2014. Further data extraction or follow-up evaluation was neither planned nor conducted.

We did not attempt to match patients in the data sources; we were interested in the naturalistic patterns of delivered care as demonstrated by each of the data sources. However, we did assess case mix; data sources for case mix factors were the registered date of birth for age, the registered postal code for socioeconomic status, and data gathered during consultations about smoking status and diabetes.

### Extracted data

Measures extracted from the claims records were ‘distribution of risk categories for dental caries, based on claims records’ (years 2011–2013) and ‘Filled-and-Missing (FM) score’ (2013). Measures extracted from the clinical records were ‘time until first restoration’ (years 2010–2014), ‘retreatment after restoration’ (2012–2014), ‘changes in periodontal-health score’ (2009–2014), ‘percentage of 18-year-olds with no tooth decay’, and ‘improvement in gingival bleeding index at reassessment’. Data for the measures ‘distribution of risk categories for dental caries, based on clinical assessment’, ‘tooth wear’, and information on smoking (yes/no) and diabetes (yes/no) on behalf of the measures ‘changes in periodontal-health score’ and ‘improvement in gingival bleeding index at reassessment’ were registered by the GDP during consultations.

The specifications of the extracted data are listed in Tables 2, 3, 4, 5, 6, 7 and Table 8.

According to the Dutch law on (bio)medical research with humans an exempt from individual written or oral participant consent applied. The project was considered to be a healthcare improvement project in which patients were included in a manner that precludes re-identification. In accordance with the rules and regulations on privacy protection and data security in the collection and exchange of data, the data was collected in the participating dental practices by an independent and trusted third party and anonymized before being analyzed. According to the same rules and regulations the identity of participating GDPs was protected in the same manner.

Birth dates were used to categorize patients in age groups for children (0–6, 7–12 and 13–17 years of age) and for adults (18–39, 40–64 and 64 and older). The postal codes of the home addresses of patients were used as indirect indicators for socioeconomic status. We used a table for socioeconomic status per postal code area from The Netherlands Institute for Social Research (SCP) to categorize patients into high, middle, or low socioeconomic status [20].

### Data analysis

The results for the measures were calculated per dental practice. The differences between dental practices were statistically tested except for the measure ‘distribution of risk categories for dental caries based on claims records insurance company’. This measure was only compared to the distribution based on clinical assessments using the Mann-Whitney U-test and Chi-square test. Case mix factors were tested on aggregated results of all dental practices. ‘Time to first restoration’ was measured as the cumulative incidence per year of age and differences between the percentage restoration-free seven-year olds between the dental practices and case mix groups were tested with Chi-square tests. ‘Distribution of risk categories for dental caries’, ‘tooth wear’ and ‘changes in periodontal-health score’ were measured in categories and calculated by dividing the number of patients per category by the total number of patients assessed for the measures concerned and expressed as percentages. Comparisons between dental practices and case mix groups were made using Kruskal-Wallis tests or Mann-Whitney U-tests depending on the number of groups. The ‘FM-score’ was calculated by the formula [(number of children and adolescents with restorations/total number of children and adolescents with at least one oral health examination) x (number of restorations/number of children and adolescents with restorations)] + [(number of children and adolescents with extractions/number of children and adolescents with at least one oral health examination) x (number of extractions/number of children with extractions)]. The ‘FM-score’ was expressed as the average number of filled and missing teeth per patient per dental practice per year, and differences between dental practices and case mix groups were analyzed with the Kruskal-Wallis test. ‘Retreatment after restoration’ was calculated as the percentage of teeth with a re-restoration within six, 12 and 18 months, and the percentage of teeth with respectively an endodontic treatment and extraction within 6 months after restoration. The denominator was the number of teeth that were restored in 2012. Comparisons were only made between dental practices using Chi-square tests.

The number of patients per case mix factor were too low for statistical tests per case mix group per dental practice; therefore we only tested case mix factors on overall results in order to detect differences in results between single case mix factors to study the influence of these factors in themselves. For the ‘FM-score’ we used the results of all patients insured by Achmea and for the other measures the results for all dental practices together.

We set the minimum number of patients to be analyzed per measure at 60; measures with less than 60 patients per dental practice were excluded. Missing data were not included in the analysis. SAS software (version 9.4) was used to calculate the results of the measures and the Kruskal-Wallis test for comparison of means of the FM-score, all other statistical tests were performed in SPSS Statistics (version 23).

### Feasibility evaluation in practice

Variations in oral health in the participating dental practices as described by the measures were discussed with GDPs during individual feedback sessions with Achmea project staff, and in a plenary meeting with the participating GDPs and Achmea project staff. In two final meetings, Achmea project staff and experts evaluated and discussed the data, results, and feasibility.

#### Feasibility testing data collection

Feasibility is dependent on the availability of data, the time and work needed to retrieve the data, and the number of patients that can be included to make results comparable [17, 21]. Retrieving data from the claims records of the insurance company required the least time and effort. However, complete datasets were only available for children up to 18 years old because oral healthcare for them is covered by legally obliged standard health insurance. Dental costs for adults are covered by voluntary supplementary insurance. The voluntary dental insurance scheme has a maximum reimbursement ceiling. Dental treatments above these maxima and dental treatments for uninsured adults are not recorded in insurance records and therefore supplementary data was assembled from the administrative databases in the dental practices. Additionally, some of the selected measures could only be assessed clinically. We evaluated the process of data collection in a meeting with the participating GDPs with use of a questionnaire. This questionnaire was developed for this study and added as a Additional file 1. Validity and reliability of the data were assessed during a meeting with the participating GDPs by showing the data and asking them whether the data seemed appropriate for their practice.

#### Face validity testing

Face validity refers to consensus between the experts that the measure represents the quality of the care being assessed [22]. We assessed face validity during the evaluation meetings with the participating GDPs and experts by answering the questions: ‘Is the measure measuring an aspect of the delivered care?’, and ‘Does the measure stimulate quality improvement?’.

#### Discriminative validity

Quality measures need to detect relevant differences between caregivers to discriminate between them [21]. This was tested by comparing the results of the participating dental practices with each other. We also tested overall results with case mix factors to detect disparities for subpopulations expected to have differences in oral health. People with a low socioeconomic status generally have poorer oral health, and age is also a critical factor [23]. Ageing is associated with increased incidence and severity of periodontal disease; smoking and diabetes are risk factors for periodontal disease [24].

#### Responsiveness

Quality measures need to detect changes over time within dental practices [21]. Due to the limited period of our study we were only able to perform baseline measurements. Responsiveness was therefore estimated and is not described in the feasibility-evaluation-in-practice section in the results.

## Results

**Fig. 1:**
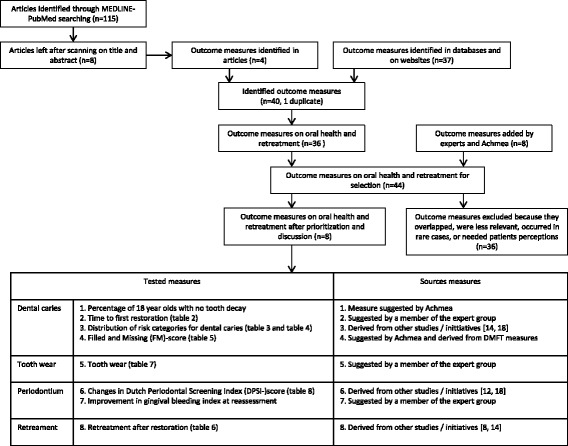
Flowchart selection outcome measures. Flowchart of the identification and selection of the outcome measures, and overview of the tested measures and their sources

The literature search revealed 115 hits (Dec 3rd 2012). After scanning titles and abstracts eight potentially relevant articles remained. Two articles were excluded because the full text was not available. From the articles, databases and websites from organizations a total of 40 outcome measures were identified. Thirty-six relevant outcome measures on oral health and retreatment were supplemented with eight measures suggested by the experts and Achmea project staff. During prioritization and discussion several measures were excluded because they were duplications, considered less relevant, or occurred in rare cases only. Measures that relied on patient perceptions were excluded because this was beyond the scope of this project. Finally, eight measures were tested in this study. Figure 1 shows the flowchart for the selection of the outcome measures.

Measures for oral health status were divided into those concerning the teeth, notably dental caries (four) and tooth wear (one), and those concerning the periodontium, notably periodontal health (one) and gingival bleeding (one). The measures on oral health were supplemented with one measure for retreatment. Of the selected measures, ‘tooth wear’ and ‘changes in periodontal-health score’ did not meet the criteria for feasibility of data collection, face validity, discriminative validity and responsiveness; main reasons were that ‘tooth wear’ was not considered sufficiently responsive and ‘changes in periodontal-health score’ was considered a controversial measure. See Tables 7 and 8.

During the data collection, we found that the available data for the selected measures ‘percentage of 18-year-olds with no tooth decay’ and ‘improvement in gingival bleeding index at reassessment’ was for less than 60 patients per dental practice and therefore were too limited to provide accurate estimates. These measures were excluded.

Tables 2, 3, 4, 5 and 6 provide the patient characteristics and results of the statistical tests for the measures describing dental caries and retreatment.

### Dental caries

#### Time to first restoration

Using survival curves, we looked at the restoration-free cumulative incidence of seven-year-olds. There were differences between the dental practices (*p* < 0.01). On average 59% of the included children had a restoration-free primary dentition at the age of seven. The range was 42% in dental practice #1 to 76% in dental practice #3. There was a difference (*p* < 0.01) in the proportion of restoration-free seven-year-olds between the different socioeconomic groups in all dental practices. In the highest socioeconomic group 67% of the seven-year-olds had a restoration-free dentition versus 45% in the lowest socioeconomic group.

#### Distribution of risk categories for dental caries based on clinical assessment

The distribution differed between dental practices (*p* < 0.01) for children and adolescents as well as for adults. For all dental practices, 45% of the children and adolescents were classified as low risk. However, in dental practice #2, 62% of the children and adolescents were classified as increased or high risk. For the adults in all dental practices 44% were classified as decreased risk, but in dental practice #3 this was 74%. Overall, younger age and a high socioeconomic status were associated with a lower risk for caries both in adults and in children and adolescents.

#### Distribution of risk categories dental caries based on claims records

For children and adolescents the patterns in distribution of risk categories was similar for both data sources in dental practice #1 and #3. The patterns in dental practice #2 differed substantially; based on clinical assessment 62% of children and adolescents were classified as high or increased risk, but based on claims records only 20% were at high or increased risk.

#### Filled-and-missing (FM) score

The FM-scores showed differences (*p* < 0.01) between the participating dental practices; the scores varied between 0.3 for dental practice #3 and 1.5 for dental practice #1. Overall, the score was 0.5 for age category 0–6 years, and 0.6 for age categories 7–12 years and 13–17 years old. The FM-score for children and adolescents with a high socioeconomic status was lower than for the other socioeconomic groups. In general, the socioeconomic status of the patients in dental practice #1 was lower than in the other dental practices, but when comparing the scores per socioeconomic group, the FM-score for dental practice #1 was found to be higher in all groups.

### Retreatment

The percentage of re-restorations differed between the dental practices for children and adolescents (*p* < 0.01) as well as for adults (*p* < 0.01). The number of re-restorations in dental practice #3 was considerably higher than in the other dental practices; within 18 months this was 15% for children and adolescents in dental practice #3 (13 out of 88 restorations) versus 0% and 1% in the other dental practices. For adults re-restoration within 18 months was 7% in dental practice #3 (140 out of 2007 restorations), whilst in the other dental practices this was lower than 2%. None of the restored teeth were endodontically treated or extracted within 6 months after restoration in children and adolescents, and there were very few in adults.

### Feasibility evaluation in practice

Table 9 summarizes the findings for feasibility of data collection, face validity, discriminative validity and estimated responsiveness for all tested measures.

**Table 9:** Feasibility evaluation of the tested measures

Measure	time to first restora-tion	distribution of risk categories for dental caries, clinical assessment	distribution of risk categories for dental caries, claims records	Filled-and-Missing score	retreat-ment	tooth wear	changes in periodontal-health score
Feasibility evaluation in practice							
Feasibility data collection							
- Availability of data and burden to retrieve the data	±	−	+ (a) ± (b)	+	±	−	−
- Number of patients that could be included	±	±	+	+	+	−	±
- Validity and reliability data	+	±	±	±	±	+	+
Face validity							
- Measure reflects aspects of quality of care	+	+	±	±	±	−	±
- Measure stimulates quality improvement	+	+	±	+	+	−	±
Discriminative validity							
- Measure shows differences between dental practices	+	+	+	+	+ (c) − (d)	±	+
- Measure shows differences after case mix correction	+	+	±	+	n/a	±	−
Responsiveness							
- Measure detects changes in time	±	±	±	+	+	−	±
*+ = positive judgment; ± = judged as doubtful or needs to be tested yet; − = negative judgment* *(a) children and adolescents* *(b) adults* *(c) re-restoration* *(d) endodontic treatment and extractions*							

#### Feasibility of data collection

##### Dental caries

Due to the small numbers per birth cohort for the measure ‘time to first restoration’ the data for several birth cohorts were combined. Data collection for the ‘FM-score’ and ‘distribution of risk categories for dental caries based on claims records was feasible for children and adolescents because full oral healthcare services are covered by mandatory insurance. For adults the data for ‘distribution of risk categories for dental caries’ was incomplete because of the limited reimbursements in the additional, optional health insurance. For ‘Distribution of risk categories for dental caries based on clinical assessment’ the number of children and adolescents per dental practice were low within the measurement period. The assessment instrument was used in different ways by the GDPs; GDP #2 counted every initial lesion, while the other dental practices only included lesions that needed restoration. There were also differences in the use of diagnostic methods as loupes, professional cleaning before examination and bitewing radiographs, which leads to higher detection rates of caries lesions. GDP #2 did not agree that the pattern of his data were representative for this measure, while the other GDPs did agree.

##### Retreatment

An advantage of the extraction of data on retreatment from the patient files is the addition of surfaces to tooth numbers. However, the surfaces were not recorded in a standard way and sometimes more in-depth codes were added. Therefore comparisons of the surfaces retreated were not feasible, and retreatment was calculated per tooth and not per restoration. GDP#3 did not think the patterns of his data were representative and found numerous reasons after checking them. Some restorations failed in teeth with an indication for extraction. Most of the retreatments in children and adolescents were caused by caries in a different surface, by rebonding orthodontic retainers and retreatment of restorations placed in a bloody setting due to trauma. Most retreatments in adults were expected, reasons were: repairs in tooth wear patients with extensive dental rehabilitations, restoration before and after endodontic treatment and stepwise excavation and restoration procedures.

#### Face validity

##### Dental caries

The findings for ‘FM-score’, ‘time to first restoration’ and ‘distribution of risk categories for dental caries based on claims records insurance company’ were influenced by the treatment indications for restorative interventions from the GDP and the used diagnostic methods. Feedback information on these measures enabled GDPs to mutually discuss their treatment approach and best practices for preventive actions, to potentially stimulate quality improvement. Risk assessments were considered helpful in the development of a structured treatment approach per risk category. The measures were viewed as providing useful feedback on the degree of success in preventing and treating caries.

##### Retreatment

There was a lot of discussion on the interpretation of this measure. Scores are influenced by patient characteristics such as bruxism or self-care, and by the treatment approach of the GDP. Some GDPs excavated stepwise to prevent endodontic treatment or monitored a carious lesion in a different surface of the tooth, whereas others chose to treat the whole tooth at once. Comparisons of the outcomes over time could potentially provide a better basis to evaluate treatment approaches, restoration materials and restoration techniques. Unexpected outcomes stimulated GDP#3 to search for the cause of these outcomes and to compare treatment approaches to other GDPs.

#### Discriminative validity

##### Dental caries

The ‘FM-score’ showed considerable treatment variation between the dental practices. Due to the high caries risk of their population, dental practice #1 filled carious lesions at an earlier stage of the caries process than the other dental practices in the expectation of preventing endodontic treatments. The differences in ‘distribution of risk categories for dental caries based on clinical assessment’ were at least partially caused by different use of diagnostic methods and assessment criteria. The dental practices in our study had apparent differences in their case mix, but it was not possible to correct for case mix factors given the low numbers per case mix factor subgroup.

##### Retreatment

The percentage of re-restorations within 18 months after restoration showed discriminative validity. But, there was no discriminative validity for endodontic treatments and extractions within 6 months after restoration; these retreatments were rare.

## Discussion

We selected eight measures on oral health for testing in practice. Four measures were evaluated as feasible and considered to be relevant measures as proxy for oral health status. These were ‘time to first restoration’, ‘distribution of risk categories for dental caries’, ‘filled-and-missing score’ and ‘retreatment after restoration’. The measure ‘tooth wear’ was not considered sufficiently responsive and ‘changes in periodontal health score’ was considered a controversial measure. The measures ‘percentage of 18-year olds with no tooth decay’ and ‘improvement in gingival bleeding index at reassessment’ were not feasible due to the limited numbers of patients for these measures per dental practice.

The feasible measures on dental caries and retreatment were judged as having face validity and discriminative validity. Information on these measures provided relevant, valid and important feedback for GDPs with a potential to improve quality in these aspects of oral healthcare. Fine tuning of the measures, testing over a longer period in a larger number of dental practices, psychometric reproducibility, multivariate testing and further research on the responsiveness are necessary. Further research is also required on the relationship between outcomes, patient populations and treatment approaches of GDPs before they can be considered as measures that truly describe improved health outcomes as a direct result of the oral healthcare provided.

### Considerations for feasible measures

The measures on dental caries and retreatment need some fine tuning.

#### Time to first restoration

Outcomes on dental caries may be biased by variation in the treatment approach of the primary dentition. In the Netherlands substantial differences in treatment approaches for caries in the primary dentition exist [1]; these vary from restoring every cavity to non-operative methods like removing undermined enamel combined with instructions to improve dental hygiene. Separate measures are needed for the primary and permanent dentition for a better understanding of the variation in the provision of oral healthcare.

#### Distribution of risk categories

Originally this measure was ‘changes in distribution of risk categories for dental caries’; therefore this required a second measurement after, say, 1 year which was not feasible as part of this study. The optimal period to reassess the caries risk was the subject of discussion. In the study of Harris et al. a period of 1 year was responsive, but GDPs suggested to take a longer time than a year, because transition to a lower risk category may take more time [18]. Clinical assessment of risk categories requires clear instructions for use of the assessment instrument. The use of a caries classification system is necessary to monitor development and progression of caries lesions and detect changes over time. Feasibility would potentially improve if automatic risk categorization could be built into the software systems for dental practices. Harris et al. based the assessment of risk categories on the social history, medical history and clinical examination on dental caries and periodontal health. Despite the broad measure they concluded that the distribution of patients in risk categories per practice was one of the most useful piloted measures [18].

#### FM-score

Separate scores are needed for the primary and permanent dentition. Discussions with practitioners are required in addition to the data; insight on GDPs’ restoration thresholds for carious lesions and the treatment approaches for the primary dentition are needed to be able to interpret the results.

#### Retreatment

If retreatment is only measured per tooth the administrative burden can be lowered by using claims records from the insurance company. Since only ‘retreatment after restoration’ showed discriminative validity, the measure ‘retreatment’ can be changed to ‘re-restoration of teeth’. The Australian Council on Healthcare Standards [8] used the measure ‘restorative treatment – teeth retreated within 6 months’; based on our results for adults, this period may be too short.

‘Time to first restoration’ is a proxy for the period of good oral health. The ‘FM-score’ shows a yearly cross section of the average number of restorations and extractions, and ‘changes in distribution of risk categories for dental caries’ provides results for a cohort. The differences between clinical assessed distributions of risk categories for dental caries and the other measures for dental caries can indicate the effects of prevention. The higher caries risk based on claims records in dental practice #3 can be explained by the high number of re-restorations, and should be taken into account in the interpretation of measures based on claims records.

### Other measures

‘Tooth wear’, and ‘changes in periodontal-health score’ did not pass the feasibility evaluation in their current form. It is not common practice to measure and record ‘tooth wear’ in daily practice and it appeared that this measure had very limited effects on the actions of the GDP. Although measuring and recording of the Dutch Periodontal Screening Index (DPSI-index) is a mandatory part of a routine oral examination in the Netherlands [19], data available from the clinical records was limited. In particular, data were not recorded when there were no changes, or when patients were treated for periodontal diseases. This is consistent with the findings of an earlier study in the Netherlands in 2012 where only 49% of GDPs measured and recorded the DPSI [12]. There was considerable discussion about the meaning of this measure and the value of the DPSI-score for assessment of the severity of periodontal disease. However, the DPSI is the only accepted measuring instrument in the Netherlands to assess periodontal health and treatment need. Further clarification and agreement of the professional approach is required to determine the meaning and eligibility of the DPSI-score as a measure for oral health status.

### Quality of care

This study is a starting point for the creation of transparency in the provision of oral healthcare services related to oral health outcomes. We found treatment variation between dental practices for some relevant measures. This probably means that not every patient receives optimal oral healthcare [1]. More clinical guidelines, widely accepted by the profession, are needed; these may then provide a basis for valid and reliable process measures. When apparently unwarranted variation is found in the presence of endorsed professional clinical guidelines or when evidence-based treatment choices are not clear, peer group discussions may be helpful in promoting healthcare quality improvement [3]. Moreover, measures on oral health outcomes as we have described can provide a focus for such discussions. We found the clinical assessments, feedback and discussions on the presented measures in itself were seen as beneficial by the GDPs. In addition, the presented measures stimulated the GDPs to evaluate their treatment approaches and reflect on their outcomes, especially when the results were not what they expected. This could potentially lead to changes in clinical practice, preventing or slowing the need for future restorations and extractions.

### Barriers and recommendations

Our approach is potentially generalizable to all countries where patients regularly visit the same dental practice. Clinical assessments during consultations are possible everywhere and clinical records, after adjusting the recording system if necessary, can be used for data extraction in situations with funding systems other than fee per item.

For discussions on quality of care it is necessary that data for measures are available. It appears there is scope for improvement in the clinical records of the dental practices [25]. Record keeping sometimes serves more for financial administration than a full clinical record to support ongoing clinical care. Unambiguous definitions and assessment methods for measures also need to be addressed [12].

There is an opportunity for software suppliers to build standard formats for simple recording of data in the IT systems used by dental practices. Data extraction from clinical records is very time consuming [12] and, depending on the health care system, claims records of an insurance company may only provide information for part of the patient population of the dental practice. A national system which automatically records a standardized dataset including standardized diagnostic codes from dental practices, would potentially provide for more accurate information on oral health treatments and outcomes [4, 6, 17]. Such a system could form the basis of a valuable feedback system for GDPs, a basis for scientific data for further research on quality of care, information for patients, and information for policy makers on oral health outcomes.

For discussions on quality of care, an open and safe environment created by trained educational facilitators, and an intention to learn are important conditions [7]. If GDPs are asked to provide data for external evaluation of the quality of their care, there might be a risk of patient selection and bias. As such, we did not intend to use measures for normative purposes; our intention was to inform and help GDPs in reflections and discussions about their oral healthcare service deliveries.

## Conclusions

The evaluated measures ‘time to first restoration’, ‘distribution of risk categories for dental caries’, ‘filled-and-missing score’ and ‘retreatment after restoration’, were considered valid and relevant measures and a proxy for oral health status. As such, they improve the transparency of oral health services delivery that can be related to oral health outcomes, and may serve to improve these oral health outcomes after further development. As yet, these measures may inform discussions on quality of oral healthcare.

## Additional file

Additional file 1: Questionnaire evaluation process data collection. (DOCX 16 kb)

## Abbreviations

DMFT/dmft: Decayed, missing and filled permanent/primary teeth
DP: Dental practice
DPSI: Dutch Periodontal Screening Index
FM-score: Filled-and-missing score
GDP: General dental practitioner
