# Supplementary material for: Outcome measures for oral health based on clinical assessments and claims data: feasibility evaluation in practice
Source: BMC Oral Health. 2017 Oct 5;17:125. doi: 10.1186/s12903-017-0410-5 (PMC5629757; doi:10.1186/s12903-017-0410-5)
Supplement: Additional file 1: — Questionnaire evaluation process data collection. (DOCX 16 kb) [file 12903_2017_410_MOESM1_ESM.docx]

**Additional file 1 - Questionnaire evaluation process data collection**

***Questionnaires clinical assessments***

1. How was the process of receiving the questionnaires for the clinical assessments?
2. Were the questionnaires clear? Were all options covered by the possible answers?
3. How was the data collection in patients?
4. How was the process of delivering data to the trusted third party?

***Data collection from patients files***

1. How was the process of the data collection from the patients files?
2. How were the contacts with the external party that extracted the data from the patients files?

***General questions***

1. If we would start all over again, what should be done differently
   1. by Achmea?
   2. by yourself?
   3. regarding the process (cooperation with the trusted third party, completing the questionnaires for the clinical assessments, getting informed consent from patients, etcetera)?
   4. regarding the content (did we ask the right questions?)?
2. Do you have any additional remarks?
